# Metastasis in context: modeling the tumor microenvironment with cancer-on-a-chip approaches

**DOI:** 10.1242/dmm.033100

**Published:** 2018-03-01

**Authors:** Jelle J. F. Sleeboom, Hossein Eslami Amirabadi, Poornima Nair, Cecilia M. Sahlgren, Jaap M. J. den Toonder

**Affiliations:** 1 Microsystems Group, Department of Mechanical Engineering, Eindhoven University of Technology, Gemini-Zuid, Groene Loper 15, 5612AZ, Eindhoven, The Netherlands; 2 Soft Tissue Engineering & Mechanobiology, Eindhoven University of Technology, Gemini-Zuid, Groene Loper 15, 5612AZ, Eindhoven, The Netherlands; 3 Institute for Complex Molecular Systems, Eindhoven University of Technology, Gemini-Zuid, Groene Loper 15, 5612AZ, Eindhoven, The Netherlands; 4 Turku Centre for Biotechnology, Åbo Akademi University, Domkyrkotorget 3, FI-20500, Turku, Finland

**Keywords:** Cancer-on-a-chip, Microfluidics, Tumor microenvironment, Cancer, Metastasis

## Abstract

Most cancer deaths are not caused by the primary tumor, but by secondary tumors formed through metastasis, a complex and poorly understood process. Cues from the tumor microenvironment, such as the biochemical composition, cellular population, extracellular matrix, and tissue (fluid) mechanics, have been indicated to play a pivotal role in the onset of metastasis. Dissecting the role of these cues from the tumor microenvironment in a controlled manner is challenging, but essential to understanding metastasis. Recently, cancer-on-a-chip models have emerged as a tool to study the tumor microenvironment and its role in metastasis. These models are based on microfluidic chips and contain small chambers for cell culture, enabling control over local gradients, fluid flow, tissue mechanics, and composition of the local environment. Here, we review the recent contributions of cancer-on-a-chip models to our understanding of the role of the tumor microenvironment in the onset of metastasis, and provide an outlook for future applications of this emerging technology.

## Abstract (precis)

**Summary:** This Review evaluates the recent contributions of cancer-on-a-chip models to our understanding of the tumor microenvironment and its role in the onset of metastasis. The authors also provide an outlook for future applications of this emerging technology.

## Introduction

**Fig. 1.**
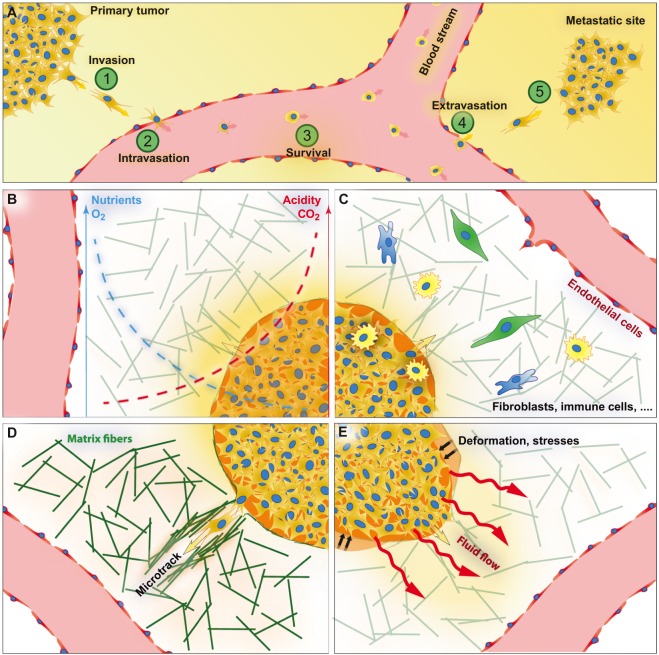
**Metastasis and the TME.** (A) The five steps of metastasis. (1) Invasion: cancer cells escape from the primary tumor into the surrounding stroma. (2) Intravasation: cancer cells cross the vessel wall and enter the circulation. (3) Survival: cancer cells survive in the circulation. (4) Extravasation: cancer cells exit the vessel and seed at a distant site after crossing the vessel wall. (5) Secondary tumor development. (B) Biochemical cues. Oxygen and nutrient levels are lower, and acidity and carbon dioxide levels are higher, within the tumor. (C) Cellular cues, from cells such as fibroblasts, immune cells and endothelial cells (ECs). (D) The extracellular matrix (ECM). The structure and biochemical properties of the ECM fibers (green lines) is heterogeneous in the TME. (E) Mechanical cues, including interstitial fluid pressure and flow, and tissue stresses and deformations.

For decades, researchers studying cancer have been focusing mainly on the genetic origin of the disease, which has led to major advances in cancer detection and treatment. Despite the increasingly effective therapeutic approaches, cancer is still one of the deadliest diseases in the world, accounting for nearly 1 in 6 of all deaths worldwide [WHO cancer fact sheet (http://www.who.int/mediacentre/factsheets/fs297/en/)]. A main challenge in treating cancer is that most deaths are not caused by the primary tumor, but by secondary tumors that form through metastasis. In this step-wise process, cancer cells go through invasion, intravasation and extravasation (see Box 1 for a glossary of terms) to ultimately form a secondary tumor, as detailed in Fig. 1A. However, we only partially understand the full complexity of the metastasis process (reviewed in Joyce and Pollard, 2009).

Box 1. Glossary **Amoeboid migration:** a mode of migration where cancer cells migrate with a low level of cell–matrix interactions and maintain a rounded, less protrusive morphology (Friedl and Alexander, 2011). This choice of migration depends on the cell type and the TME, and does not require EMT.

**Angiocrine signaling:** signals produced by endothelial cells (ECs) that can affect cancer cell behavior.

**Angiogenesis:** the process through which new blood vessels form in the TME, sprouting from existing vessels.

**Basement membrane (BM):** a type of pericellular matrix that is in close contact with the epithelial tissue.

**Cancer-associated fibroblasts (CAFs):** activated fibroblasts in the TME, with extensive roles in cancer.

**Epithelial-to-mesenchymal transition (EMT):** the transition through which cells obtain a more migratory and mesenchymal phenotype, with fewer cell–cell and more focal adhesion sites (Friedl and Alexander, 2011).

**Extracellular matrix (ECM):** the non-cellular fibrous regulatory support structure of most tissues. In this Review, ECM solely refers to the collagen-I-rich interstitial matrix.

**Extravasation:** when cancer cells leave the circulation by crossing the vessel wall to enter a metastatic niche.

**Intravasation:** when cancer cells cross the vessel wall to enter the circulation.

**Invasion:** when cancer cells break through the BM and invade the stromal tissue surrounding the tumor.

**Matrix metalloproteinases (MMPs):** a family of proteolytic enzymes capable of degrading the ECM, secreted by or membrane-tethered to cancer and stromal cells (reviewed in Lynch and Matrisian, 2002).

**Mesenchymal migration:** a mode of migration in which cancer cells migrate with strong cell–matrix interactions and an elongated, more protrusive morphology (Friedl and Alexander, 2011). This choice of migration depends on the cell type and the TME, and generally requires EMT.

**Microfluidic chip:** a device that contains small channels, with cross-sectional dimensions typically below 1 mm. Different channel arrangements and control methods enable very accurate control of fluid flow, (shear) forces and pressure (reviewed in Whitesides, 2006).

**Paracrine signaling:** signals produced by cells to induce changes in the neighboring cells in their microenvironment.

**Solid stress:** the stresses within the tumor resulting from high proliferation of cancer cells and ECM stiffening.

**Spheroids:** spherical three-dimensional (3D) aggregates composed of proliferating cancer cells.

**Tumor-associated macrophages (TAMs):** the most abundant immune cells present in the TME.

**Tumor microenvironment (TME):** the environment that immediately surrounds the cancer cells within a tumor, comprised of biochemical signals, different cellular populations, the ECM and mechanical cues.

**Warburg effect:** the phenomenon whereby glucose consumption is elevated in cancer cells. This difference in the metabolism between cancer cells and healthy cells is due to cancer cells relying more on inefficient glycolysis for energy production, whereas healthy cells generally rely on oxidative phosphorylation (respiration) in the mitochondria.

We do know that metastasis is not only driven by intrinsic factors such as the (epi)genetic characteristics of the cancer cells, but is also critically affected by cell-extrinsic factors mediated by the tumor microenvironment (TME; Box 1; reviewed in Hanahan and Weinberg, 2011). In this Review, we focus on the role of the TME in driving tumor invasion, angiogenesis (Box 1) and intravasation into the vasculature, thereby initiating cancer cell dissemination throughout the body. A major challenge in understanding the role of the TME is that a systematic analysis of the influence of individual TME components is still very difficult to achieve.

Current experimental approaches to study cancer invasion are based on *in vitro* two-dimensional (2D) or 3D cell cultures, complemented by *in vivo* animal models using human cell lines or patient-derived xenografts (reviewed in Alemany-Ribes and Semino, 2014; Choi et al., 2014). These approaches have been important for our current understanding of cancer, but they also have some limitations. Most importantly, growing cells in 2D culture models does not capture the 3D nature of tumors and leads to deviating cellular behavior (reviewed in Weigelt et al., 2014). Current 3D models, such as cancer spheroids (Box 1) and 3D hydrogel cultures, have greatly improved upon this, and are often compatible with the methodologies for 2D models, enabling the use of conventional experimental read-outs. However, a disadvantage of current 3D models is the static (non-flow) nature of these models, which limits the researchers' control over local biochemical gradients, but is also very different from the vascularized *in vivo* tissue. Additionally, most 3D models are mono-cellular and do not include other cell types typically found in the TME. Animal models intrinsically contain a more complete representation of the *in vivo* TME complexity, yet their use is less straightforward: they are generally inefficient, expensive and not always a good representation of the human (patho-)physiology.

To complement the current research models and overcome some of their limitations, several groups are developing and using so-called cancer-on-a-chip models (CoC; Box 2). In this Review, we discuss the current status of CoC research, particularly in relation to our current knowledge about the role of the TME in the onset of metastasis. We briefly revisit the TME as we understand it from traditional *in vitro* and *in vivo* research models, after which we review the contributions of CoC models in more detail. Furthermore, we highlight the most important outstanding challenges regarding the interactions between cancer cells and their environment, and discuss how future developments in CoC technology could contribute to tackling these challenges.

Box 2. Cancer-on-a-chip Cancer-on-a-chip (CoC) models are based on microfluidic chips with micrometer- to millimeter-sized compartments and microchannels that enable controlled fluid transport. The compartments can be used to reproducibly create a niche in which ‘mini-tumors’ can grow, develop and interact within their own specified microenvironment, similarly to human tumors (reviewed in Lee et al., 2016; Portillo-Lara and Annabi, 2016). Their small size allows the cellular and matrix composition, local biochemical gradients and mechanical forces, such as shear and stretch, to be highly controlled. These compartments are optically accessible for live observation, as most chips are made from polydimethylsiloxane (PDMS) using the process of soft lithography (reviewed in Xia and Whitesides, 1998). PDMS is a soft, transparent silicone material that is permeable to gases, enabling O_2_ and CO_2_ equilibration. Additionally, all microfluidic devices work with small reagent volumes, which reduces the experimental costs.

**Fig. 2.**
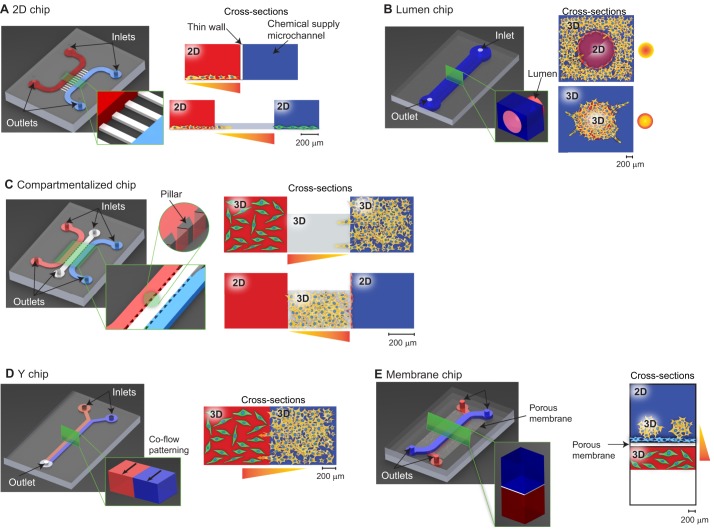
**Cancer-on-a-chip (CoC) designs with different cell culture options.** The complete chips are typically a few cm in size: (A) 2D chip. Single- or multi-chamber 2D culture devices with a controlled solute gradient. In this type of chip, cancer cells are typically exposed to a gradient of a solute, such as oxygen, while their viability or migration is measured. (B) Lumen chip. A patterned 3D matrix is used to form lumens or tumor compartments. This design is typically used to model blood vessels in tumors, or to tightly pack cancer cells in a cylindrical compartment. (C) Compartmentalized chip. In this device, pillars are used to separate microchannels in which cell culturing is possible in both 2D and 3D. This type of chip is very versatile, allowing the user to pattern different matrix materials and cells in a controlled manner. (D) Y chip. Parallel matrix compartments patterned by co-flow. This chip type resembles the compartmentalized chip, as it enables matrix patterning, but is slightly less versatile in its patterning possibilities. (E) Membrane chip. A co-culture device with stacked microchannels separated by a porous membrane. One of the interesting features of these devices is that a 3D culture is created only in part of the microchannel, with the rest empty to refresh the media. This multi-layered chip type was originally developed to mimic the endo- and epithelial cell layers found in the lung. In all images, cancer cells are indicated in yellow, additional cell types in red, green or blue, and solute gradient directions as yellow-red gradients.

Different types of CoC models exist, as detailed in Fig. 2. They contain microfluidic compartments to culture cells, either on a flat substrate (in 2D chips) or in a 3D matrix (in lumen, compartmentalized or Y chips), or in a double layer separated by a porous membrane (in membrane chips). Depending on their design, different cues from the TME can be modeled and accurately controlled in these chips. These properties make CoC devices an excellent tool for studying the interactions between cancer cells and their microenvironment.

## The tumor microenvironment

Here, we categorize the factors that define the TME into four groups (Fig. 1B-E): (1) biochemical cues, or the soluble factors affecting cancer cells; (2) other cell types in the TME, such as immune cells and fibroblasts; (3) the extracellular matrix (ECM; Box 1); and (4) mechanical cues, such as interstitial fluid flow. We briefly review what is known and unknown about the significance of these factors for cancer invasion, angiogenesis and intravasation on the basis of current research models, such as conventional *in vitro* cell cultures and animal models.

### Intrinsic biochemical changes in the tumor microenvironment

In solid tumors, solute transport is limited, but energy demands and waste generation are high. This discrepancy results in gradients arising throughout the tumor (Fig. 1B). Here, we highlight the solutes that are known to affect cancer cells: oxygen and metabolic products.

#### Oxygen gradients and hypoxia

When exposed to hypoxic (low oxygen) conditions in the tumor, cells activate several mechanisms to avert hypoxia-induced apoptosis. One such example is angiogenesis induced via hypoxia inducible growth factor (HIF)-1-α. This transcription factor affects the expression of genes responsible for angiogenesis, cell survival, cell metabolism and invasion (reviewed in Semenza, 2003). In the context of invasion, the most direct downstream effects of HIF-1-α overexpression are the epithelial-to-mesenchymal transition (EMT; Box 1) and increased amoeboid invasion (Box 1) in epithelial cancers (Lehmann et al., 2017). Additionally, hypoxia can affect cancer cell invasion by activating other stromal cells in the TME and by remodeling the ECM (reviewed in Semenza, 2016).

#### Cancer cell metabolism and extracellular acidity

A distinct difference between cancer and healthy cells is found in their metabolism: due to the above-mentioned limited transport of solutes within a tumor, cancer cells rely on less efficient pathways to generate energy. This difference, referred to as the Warburg effect (Box 1), causes an elevation in both the extracellular acidity and lactate concentration. There is growing evidence that this increases the invasiveness of cancer cells (reviewed in Kato et al., 2013). Moreover, elevated extracellular acidity has been shown to negatively affect the healthy tissue surrounding breast, prostate and colon tumor xenografts, making it more susceptible to cancer cell invasion (Estrella et al., 2013; Gatenby et al., 2006; Rofstad et al., 2006). Lactate was found to have similar effects on carcinoma cells *in vitro* (Goetze et al., 2011).

Oxygen, extracellular acidity and lactate clearly have links to metastasis, but studying the impact of these biochemical gradients using conventional approaches is still challenging.

### Cellular components of the tumor microenvironment

This section highlights the most studied cells in the TME: inflammatory cells, cancer-associated fibroblasts (CAFs; Box 1), and endothelial cells (ECs; Fig. 1C).

#### Inflammatory cells

Cancer cells and TME stromal cells recruit inflammatory cells from the circulation (reviewed in Balkwill and Mantovani, 2001; Coussens and Werb, 2002; Mantovani et al., 2008). These cells can have both tumor-suppressing and -promoting effects (Coussens and Werb, 2002; Mantovani et al., 2008). Among the immune cells in the TME, macrophages are the most abundant (as reviewed in Hu et al., 2016), and we discuss them in more detail here.

Tumor-associated macrophages (TAMs; Box 1), which derive from recruited circulating monocytes (reviewed in Hu et al., 2016; Mantovani and Sica, 2010), can have two phenotypes: M1 and M2. Depending on this phenotype, which is highly influenced by cues from the TME, TAMs can have contrasting roles in cancer (reviewed in Lewis and Pollard, 2006; Mosser and Edwards, 2008; Sica et al., 2008). M1 macrophages generally have pro-inflammatory tumor-suppressing properties in the early stages of cancer, but they polarize towards the M2 phenotype as the tumor progresses. These M2 TAMs secrete cytokines and growth factors to suppress anti-tumor inflammatory activities (reviewed in Mantovani et al., 2002; Sica et al., 2008). In addition, they can directly promote invasion, secrete pro-angiogenic factors, such as vascular endothelial growth factor (VEGF) (Lin et al., 2006), and remodel the ECM by expressing and activating matrix metalloproteinases (MMPs; Box 1) (Sangaletti et al., 2003).

The pro-tumor activity of the TAMs makes them a suitable target for anti-tumor therapies (reviewed in Belgiovine et al., 2016; Mantovani and Allavena, 2015). For example, M2 TAMs can be switched to the M1 type to trigger an anti-tumor response of the immune system (Buhtoiarov et al., 2011; Rolny et al., 2011). However, therapeutic interventions can also push TAMs towards a more tumor-supporting function (Dijkgraaf et al., 2013), so better models are needed to increase our insight in the effects of drugs on TAMs. In addition, work in conventional model systems revealed much about the role of TAMs, but it is important to recognize that most of our current knowledge is based on mouse models. Species-specific differences might affect TAM recruitment and activation mechanisms (reviewed in Ostuni et al., 2015), thereby hampering the translation of this knowledge into the context of human cancer.

#### Cancer-associated fibroblasts

CAFs are extremely abundant in the tumor stroma. They are recruited and activated by cancer cells (reviewed in Xouri and Christian, 2010). In healthy tissues, fibroblasts are responsible for ECM deposition, regulating epithelial differentiation, inflammation and wound healing (reviewed in Cirri and Chiarugi, 2012; Darby and Hewitson, 2007; Parsonage et al., 2005). In tumors, CAFs have been shown to enhance cancer cell proliferation, invasion and angiogenesis (reviewed in Kalluri and Zeisberg, 2006; Kuzet and Gaggioli, 2016; Räsänen and Vaheri, 2010; Shimoda et al., 2010). Together with cancer cells, CAFs reorganize the ECM, potentially contributing to most of the exogenous EMT stimuli during cancer invasion (reviewed in Bhowmick et al., 2004; Cirri and Chiarugi, 2012). As they are directed by pro-fibrotic signals from the cancer cells, they partly govern the volume and composition of the tumor stroma (Kalluri and Zeisberg, 2006). CAFs appear to be very similar to activated fibroblasts in wound healing, but it is unclear whether this means that they are the same cell type, or whether CAFs acquire properties that are unique to the TME (reviewed in Bierie and Moses, 2006; Darby and Hewitson, 2007). Furthermore, the role of mechanotransduction in CAF activation is not yet fully understood (Kuzet and Gaggioli, 2016).

Because CAFs are genetically more stable than cancer cells and play an important role in cancer metastasis, they are an interesting target for cancer therapy (Cirri and Chiarugi, 2012). For example, the bilateral signaling between cancer cells and CAFs can be inhibited to prevent cancer invasion (reviewed in Tao et al., 2017).

#### Endothelial cells

In solid tumors, angiogenesis is a process that accompanies and supports tumor growth, and is characterized by the development of heterogeneous, chaotic, distorted and leaky vessel networks (Hanahan and Weinberg, 2011; Nagy et al., 2010). The new vessels provide the tumor with oxygen, nutrients and waste disposal, and facilitate cancer cell intravasation. As such, targeting angiogenesis to oppose cancer progression has received considerable attention, and trials with angiogenesis-inhibiting drugs are in progress. Angiogenesis can be induced via angiogenic factors, such as VEGF-A and angiopoietin-2 (ANGPT2) (reviewed in Semenza, 2013). Similarly, the formation of lymphatic vessels can be induced by VEGF-C and VEGF-D (reviewed in Van Zijl et al., 2011). Recently, ECs have been proposed to directly affect cancer progression through angiocrine signaling (reviewed in Butler et al., 2010) and paracrine signaling (Cao et al., 2014) (Box 1). The idea of the involvement of angiocrine signaling in cancer is supported by *in vitro* data showing that ECs enhance the metastatic potential of cancer cells (Ghiabi et al., 2014), but the *in vivo* relevance of this finding is not yet clear. Additionally, the mechanisms that underlie endothelial barrier transmigration in the complex TME are not yet fully understood. It is important to recognize that ECs and other TME cells do not act in isolation, but are continuously in contact with their surroundings. For example, ECs can dramatically affect the biochemical gradients in the TME, or the supply of inflammatory cells, by altering blood flow.

### The extracellular matrix in cancer

The ECM is the non-cellular component in all tissues and organs that provides cells with chemical and mechanical support (Fig. 1D; reviewed in Bissell et al., 1982; Frantz et al., 2010). Dynamic cross-talk between the cells and the ECM maintains tissue homeostasis (Bissell et al., 1982). In tumors, however, microenvironmental stimuli, such as hypoxia and solid stresses (Box 1), drive excessive matrix remodeling, as illustrated in Fig. 1D (reviewed in Lu et al., 2012). This remodeling is a result of basement membrane (BM; Box 1) and interstitial ECM degradation by overexpressed matrix-degrading enzymes, such as MMPs (reviewed in Deryugina and Quigley, 2006; Vihinen and Kähäri, 2002), by the increased deposition of new matrix components and by lysyl oxidase (LOX)-dependent crosslinking of ECM proteins (Cox et al., 2013).

Remodeling leads to changes in the physical properties of the ECM, such as increased stiffness, which plays an important role in cancer progression (reviewed in Butcher et al., 2009; Kumar and Weaver, 2009; Paszek and Weaver, 2004). Increased matrix stiffness has been linked to increased cell traction forces that fuel cell migration (reviewed in Paszek et al., 2005), to malignant transformation and to activation of the EMT program (Leight et al., 2012; Paszek et al., 2005). Additionally, tumor growth leads to thinning and softening of the BM, which could help cancer cells to invade through this barrier (Butcher et al., 2009; Kumar and Weaver, 2009; Paszek and Weaver, 2004).

Like ECM stiffness, ECM topography is highly dynamic. Aligned ECM fibers and weakened microtracks are a typical sign of invasive tumors (Friedl and Wolf, 2008). Furthermore, a remodeled matrix topography affects the stability and bioavailability of ligands on the ECM fibers, as well as the accessibility of growth factors and cytokines, thereby influencing tumor development (reviewed in Egeblad et al., 2010; Hynes, 2009).

Due to the complexity of the cancer-cell–ECM interactions, understanding the reciprocal relationship between the matrix and cancer cells is still challenging: ECM remodeling can promote invasion, but is itself also induced by invasion (reviewed in Kumar and Weaver, 2009). New therapies targeting the ECM require a better understanding of the cell–ECM interaction. For example, a deeper insight on this interaction can result in more effective therapies that inhibit the degradation and production of the ECM during cancer invasion (Chen et al., 2017; Cox et al., 2013). Within the TME, the ECM also indirectly relays mechanical cues to cancer cells, such that changes in stiffness and topography can change how different mechanical cues affect the tumor, as discussed below.

### Mechanical cues in the tumor microenvironment

Mechanical cues, such as fluid pressure, shear stress, solid stress (reviewed in Koumoutsakos et al., 2013; Kumar and Weaver, 2009) and tissue level deformations, especially relevant in tissues that are subject to dynamical loading, such as the colon (Whitehead et al., 2008), can affect cancer cells.

In most solid tumors, the interstitial fluid pressure (IFP) is elevated due to the leaky vasculature and the increased stiffness of the ECM (reviewed in Shieh and Swartz, 2011). Generally, an elevated IFP leads to an increase in the interstitial flow velocity, especially at the tumor–stroma interface, which has been linked to increased cancer cell invasion in patients (Hompland et al., 2014).

Other mechanical cues originate from deformation at the tissue level. An example of this is the cyclic tensile strain in the lung, which occurs during breathing and has been shown to affect the drug responsiveness of lung cancer cells (Hendricks et al., 2012). Although direct therapeutic intervention in mechanical cues is not straightforward, indirect methods to affect tissue stresses and IFP, for example via LOX inhibition (Cox et al., 2013), could be employed for metastasis prevention. However, the full impact of tissue-level mechanical cues on cancer cell invasion has not been studied in detail because introducing these in an *in vitro* model is challenging.

## The contributions of cancer-on-a-chip

**Fig. 3.**
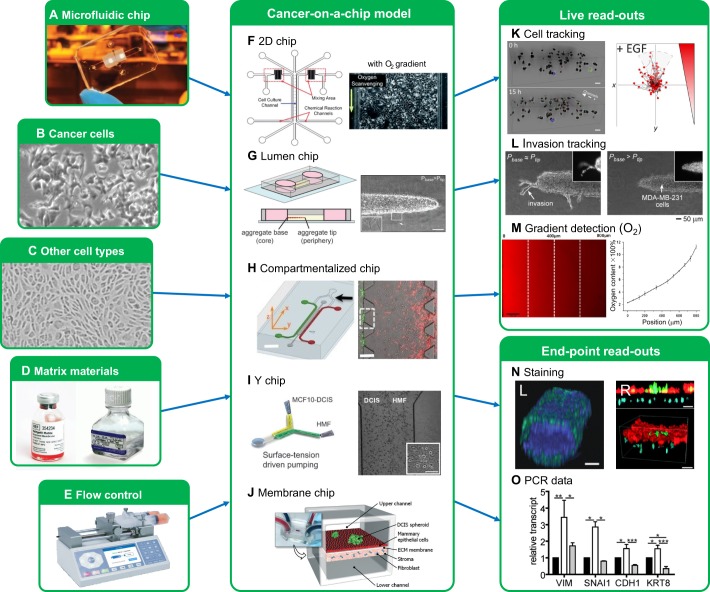
**CoC in practice.** The key input elements of CoC models are: (A) a microfluidic chip, (B) cancer cells, (C) additional cells (optional), (D) matrix materials (optional) and (E) equipment to control fluid flow, such as a syringe pump. Using these elements, the different CoC model types can be built: (F) 2D chips in which chemical gradients can be established (indicated by the arrow) [adapted from Chen et al. (2011) with permission from The Royal Society of Chemistry], (G) lumen chips (adapted from Piotrowski-Daspit et al., 2016, with permission from The Royal Society of Chemistry), (H) compartmentalized chips (Zervantonakis et al., 2012), (I) Y chips (adapted from Sung et al., 2011, with permission from The Royal Society of Chemistry) and (J) membrane chips (adapted from Choi et al., 2015, with permission from The Royal Society of Chemistry). Different experimental read-outs are possible, with some typical examples shown in K-O. The main strength of the CoC approach is that it allows continuous live monitoring of model development. (K) Individual cells (Truong et al., 2016) and (L) invasive lesions (Tien et al., 2012) can be tracked. (M) Solute levels can be tracked (adapted from Wang et al., 2015a, with permission from Springer Nature). These live read-outs can be combined with end-point read-outs, such as tissue staining [N; L, Bischel et al., 2015; R, adapted from Choi et al. (2015), with permission from The Royal Society of Chemistry], and (O) gene expression data (adapted from Piotrowski-Daspit et al., 2016, with permission from The Royal Society of Chemistry). DCIS, ductal carcinoma *in situ*; HMF, human mammary fibroblasts; *P*_base_, pressure at cell aggregate base; *P*_tip_, pressure at cell aggregate tip. Scale bars: 50 μm (G), 2 mm (H, left), 300 μm (H, right), 30 μm (I), 100 μm (K,N).

Although conventional models have significantly contributed to our knowledge of metastasis, CoC models have started to yield new insights into the role of the TME in metastasis initiation in recent years. Here, we review the contributions of CoC models for each of the TME components that we defined above. We have categorized the different CoC designs into five groups, as detailed in Fig. 2. Fig. 3 illustrates how these designs are operated in practice, showing a number of examples from the literature. Researchers generally choose between the 2D, lumen, compartmentalized, Y or membrane chips based on which TME cues they are studying. However, the basic components of a CoC remain the same: a microfluidic chip (Box 1), cancer cells, other cell types (optional), matrix materials (optional) and equipment to control fluid flow, such as a syringe pump (optional). The controlled parameters and read-out methods can differ between chip types, but common read-outs are based on cell and invasive lesion tracking, gradient sensing, staining and gene expression quantification using quantitative reverse-transcription polymerase chain reaction (RT-qPCR; Fig. 3). For an overview of the available literature in table format, we refer the reader to Table S1.

## Modeling intrinsic biochemical changes in the tumor

### Oxygen gradients and hypoxia

Different methods have been used to generate oxygen gradients based on the steady-state diffusion of oxygen from high to low concentration. A locally created balance between a source and a sink of oxygen can control both the magnitude and the direction of the gradient.

This can be achieved in 2D chips using chemicals, either inside the cell culture channel (Wang et al., 2013) or in parallel channels (Chen et al., 2011; Wang et al., 2015a). Examples of a parallel microchannel design and live oxygen detection are shown in Fig. 3F and M, respectively (Chen et al., 2011). Typical examples of oxygen-scavenging chemicals are pyrogallol combined with sodium hydroxide (NaOH), and sodium sulphite, whereas typical oxygen sources are the environment, or hydrogen peroxide (H_2_O_2_) combined with sodium hypochlorite (NaClO). A CoC approach using parallel channels could successfully determine the effectiveness of several therapeutic agents as a function of the oxygen tension, which could be useful in drug response studies. In this type of device, however, gradients remain stable as long as the chemicals are continuously refreshed to maintain the reaction, which has the downside that reaction waste is continuously produced.

Alternatively, waste-free gas-supply channels can be used as sources and sinks of oxygen. Based on this method, a gradient across a 3D hydrogel (Oppegard and Eddington, 2013) and a gradient across a compartmentalized chip with a collagen ECM and a vessel-mimicking channel (Acosta et al., 2014) could be generated. Using the compartmentalized chip, Acosta et al. determined cancer cell invasiveness as a function of oxygen concentration. Other researchers enhanced local gradient control by limiting environmental oxygen influx using impermeable layers in the device (Chang et al., 2014; Funamoto et al., 2012), and thus enabled more accurate quantification of the oxygen response. Interestingly, using a 2D chip, researchers found evidence of a direct influence of oxygen gradients on the direction of cell migration in A549 lung carcinoma cells, which tended to migrate towards lower oxygen concentrations, a process termed aerotaxis (Chang et al., 2014). Similarly, MDA-MB-231 breast cancer cells were recently found to respond to aerotaxis, but in the opposite direction, migrating towards the higher oxygen concentration. However, the 2D chip design in this study lacked oxygen control, which limited its ability to draw conclusions on the relevance of aerotaxis in this cancer cell type (Yahara et al., 2016). If aerotaxis is persistent across cancer cell types, but with different directionality, it could have a direct impact on the effectiveness of therapies, such as angiogenesis inhibition, for different cancers.

### Cancer cell metabolism and extracellular acidity

Currently, little work has focused on investigating the metabolism-related concentration gradients in CoC systems. To our knowledge, active control over acidity or acid/lactate gradients has not been shown. However, some work has been done on quantifying the concentration and distribution of metabolites inside 2D and Y chips (Walsh et al., 2009; Xu et al., 2015), highlighting that microfluidics hold the potential to advance this field.

## Modeling the cellular environment of the tumor

### Tumor-associated macrophages

Macrophage-mediated cancer cell invasion has been one of the most frequently studied applications of CoC models. Zervantonakis et al. cultured cancer cells, macrophages and ECs in a compartmentalized chip, shown in Fig. 3H (Zervantonakis et al., 2012). They observed that TAMs significantly increased the ability of cancer cells to impair the endothelial barrier and intravasate. Bai et al. used a similar design to investigate TAM-mediated activation of EMT, and found that different TAM subtypes can disperse cancer cell aggregates via different mechanisms (Bai et al., 2015). For example, they observed that a subtype of M2 macrophages could only promote cell aggregate dispersion through direct contact.

Several CoC-based publications also show that cancer cells directly affect TAMs, increasing the migration and affecting the polarization of resident macrophages (Huang et al., 2009; Zhao et al., 2015). For instance, Huang et al. used a compartmentalized chip and observed that invasive cancer cells recruited macrophages rather than migrating towards them.

These studies show that CoC devices can help us understand the activation of the TAMs, and how these macrophages enhance cancer invasion.

### Cancer-associated fibroblasts

Real-time imaging in CoC models has been used to study how CAFs affect cancer cell migration (Liu et al., 2007; Ma et al., 2010; Truong et al., 2016; Yu et al., 2016). Typically, cell tracking techniques are used to analyze cancer cell migration, as illustrated in Fig. 3K (Truong et al., 2016). For example, Liu et al. observed collective cell migration of adenoid cystic carcinoma cells into BM matrices when co-cultured with CAFs in a compartmentalized chip. This behavior was repressed when MMP expression was inhibited in both cell types, implying that MMP-mediated matrix proteolysis is critical to cancer invasion (Liu et al., 2007). In a different study, in which CAFs and cancer cells were co-cultured in a compartmentalized chip, CAFs were shown to lead the forefront of cancer cell migration into a BM matrix (Li et al., 2016). In another study, Sung et al. used a Y chip to culture non-invasive mammary ductal carcinoma cells in the proximity of fibroblasts, as shown in Fig. 3I (Sung et al., 2011). They controlled the distance between the cancer cells and fibroblasts and observed that the cancer cells' transition to an invasive phenotype depends on this distance. The same group used a hybrid lumen–compartment chip and observed the transition of non-invasive ductal carcinoma cells to an invasive phenotype only when these cells were cultured with fibroblasts (Bischel et al., 2015). In contrast, negligible cancer cell invasion was observed in a membrane chip that contained carcinoma spheroids and mammary epithelial cells in one compartment, with fibroblasts in an adjacent compartment, as shown in Fig. 3J (Choi et al., 2015). In addition, several publications showed trans-differentiation of dormant fibroblasts to activated fibroblasts when they were co-cultured with cancer cells (Gioiella et al., 2016; Hsu et al., 2011; Ma et al., 2010).

So far, these CoC models have helped us to better understand the invasion-related interactions between CAFs and cancer cells, highlighting the importance of CAFs in promoting cancer cell metastasis.

### Endothelial cells

Several CoC-based studies have focused on the interactions between cancer and ECs, mostly using hydrogel matrices. In general, these models have a gel–fluid interface that is lined with ECs to mimic a vessel wall, but their geometry varies.

For example, the previously mentioned compartmentalized chip from Zervantonakis et al. (2012) contains a rectangular channel lined with ECs. The ECs are in contact with a cancer-cell-laden collagen I matrix, between the micropillars that separate the compartments. This design has the advantage of relatively simple imaging due to the well-defined tumor-vessel boundary, and permits the subsequent introduction of other cues, such as growth factors.

More *in vivo*-like cylindrical vessels were also constructed by patterning cylindrical channels in a cancer-cell-laden collagen I matrix, and lining them with ECs (Wong and Searson, 2014). Wang et al. further developed this type of model and also incorporated a BM model by patterning the cylindrical channel with a polysaccharide microtube (Wang et al., 2015b). Although the shapes of these models are more physiologically relevant, quantification and imaging read-outs become more challenging.

Even more *in vivo-*like vessels have been designed by relying on EC self-assembly, provided that the right cues are present in the chip. Lee et al. used a multi-compartmentalized chip to drive human umbilical vein endothelial cells (HUVECs) to differentiate and self-assemble into a blood vessel inside a fibrin gel, a matrix material normally involved in wound healing (Lee et al., 2014). Nearby compartments were seeded with lung fibroblasts to provide the growth factors to induce and direct HUVEC self-assembly. These models are inherently more similar to *in vivo* vessels, but also make quantitative analysis more challenging, again illustrating the trade-off between physiological relevance and ease of analysis.

Using the models from Lee et al. (2014) and Zervantonakis et al. (2012), the effects of tumor necrosis factor alpha (TNF-α) on vessel wall permeability and invasion rate were observed live, for both breast cancer and fibrosarcoma cells. Additionally, the model described in Wang et al. (2015b) was used to demonstrate the pro-invasion effect of hepatocyte growth factor (HGF) for liver cancer cells.

In other work, intravasation into lymphatic vessels was studied using a hybrid Transwell–microfluidic system that resembled a membrane chip (Pisano et al., 2015). In this system, both luminal and transmural flow could be controlled, and both flow types were shown to have a promoting effect on the intravasation of breast cancer cells.

The main power of these methods is that they enable live observation of intravasation dynamics, such that other relevant microenvironmental factors can be systematically studied, down to the single-invasion-event level. For example, one could incorporate different ECM environments into the chips to facilitate research into the effect of ECM properties on EC resistance to cancer cell invasion.

## Modeling the cancer-cell–ECM interactions

Injectable hydrogels, mainly collagen I and Matrigel, a type of reconstituted BM, are often used as 3D matrices to support cell growth and migration in microfluidic devices (Huang et al., 2009; Shin et al., 2014; Truong et al., 2016). Recently, self-standing matrix layers, such as electrospun matrices, in a membrane chip have been developed as an alternative (Eslami Amirabadi et al., 2017). These matrices offer more mechanical stability compared to the hydrogels. When modeling cancer-cell–ECM interactions in such CoC devices, the primary read-out is usually the effect of the matrix composition on cancer invasion. For example, several studies compared various ECM compositions between Matrigel, collagen I and a mixture of both to find the most appropriate matrix to study cancer invasion (Anguiano et al., 2017; Huang et al., 2009; Sung et al., 2011; Truong et al., 2016). Sung et al., using a Y chip, observed that non-invasive epithelial cancer cells require the mixture of the both gels to grow in 3D clusters and transition to an invasive phenotype (Sung et al., 2011). In another study, focused on the cancer cell heterogeneity in breast cancer, Shin et al. used a compartmentalized chip. They observed that MCF-7 cells, an epithelial-like non-invasive cancer cell line, only follow the invasion path of MDA-MB-231 cells, a highly invasive cancer cell line, when grown in Matrigel, but not when grown in collagen I (Shin et al., 2014). In a different study, Han et al. used a compartmentalized chip to create an assembly comparable to the *in vivo* structure by aligning collagen fibers perpendicularly to a neighboring Matrigel layer (Fig. 1D). They observed that this heterogeneous interface makes the cells orient along the collagen fibers and invade into the Matrigel layer, whereas cells in a homogeneous interface did not invade the Matrigel (Han et al., 2016).

The CoC community has also devoted significant attention to visualizing ECM remodeling, for which different microscopy techniques can be used, such as second harmonic generation (SHG) (Drifka et al., 2013; Gioiella et al., 2016; Huang et al., 2009; Sung et al., 2011), fluorescence (Shin et al., 2014; Sung et al., 2011), phase contrast (Han et al., 2016; Shin et al., 2014) and scanning electron microscopy (Chaw et al., 2007). For instance, Wong and Searson used a lumen chip to image the formation of ECM microtracks that cancer cells create towards blood vessels using phase contrast and fluorescence microscopy (Wong and Searson, 2014). In the previously mentioned work by Sung et al., researchers studied the individual roles of cancer cells and fibroblasts in matrix remodeling using SHG microscopy (Sung et al., 2011).

Only a few CoC publications have studied the relationship between the mechanical properties of the ECM and cancer cell invasion. For example, Wong and Searson suggest that stiffness and pore size in the ECM can be optimized to enhance invasion by using a lower collagen concentration in dense matrices (Wong and Searson, 2014). A reverse strategy, e.g. reinforcing the weakened ECM by artificial materials, can be a therapeutic approach to prevent cancer invasion, especially in the early stages of metastasis (reviewed in Chen et al., 2017).

Current CoC platforms have helped us understand how ECM composition and its structure can affect cancer cell invasion by visualizing matrix remodeling with different imaging techniques. In spite of this progress, CoC models have much more potential to unravel cell–matrix interactions during cancer invasion, as we discuss below.

## Modeling mechanical cues in the tumor

### Interstitial fluid pressure and flow

Similarly to ECM-focused studies, lumen and compartmentalized chips have been predominantly used to investigate the effects of IFP. These CoC approaches enabled researchers to, for the first time, directly observe the response of cancer cells to IFP, and to the interstitial fluid flow (IFF) that is caused by IFP gradients.

In a lumen chip, human MDA-MB-231 breast cancer cells grown as cell aggregates in collagen I could reproducibly be subjected to an IFP gradient, with high pressure at the base of the aggregates and low pressure at the tip, and *vice versa* (Tien et al., 2012). The authors measured invasion from the cell aggregate tips as illustrated in Fig. 3L, and showed that high IFP at the base decreased the invasiveness of the cell aggregate, whereas low IFP at the base increased invasiveness. This invasion against a pressure gradient was also observed in the HepG2 and HLE liver cancer cell lines, using a collagen I matrix in a compartmentalized chip (Kalchman et al., 2013). These studies indicate that cancer cells of different types tend to invade towards regions of higher pressure, such as intratumoral blood vessels, to potentially metastasize. Interestingly, increased IFF from the tumor base to its edge seems to inhibit invasion from the tumor margin, indicating that invasion towards intratumoral blood vessels might be the dominant mechanism for metastasis *in vivo*. By combining the model from Tien et al. (2012) with other analyses, such as western blotting and RT-qPCR, both mesenchymal markers, such as Snail and vimentin, and the epithelial markers E-cadherin and keratin-8 were found to be upregulated under the invasion-inducing IFP gradient (Fig. 3G,O) (Piotrowski-Daspit et al., 2016). In this condition, cancer cells invaded collectively against the imposed IFP gradient, explaining how the upregulated epithelial markers related to cell–cell contact. The upregulation of mesenchymal markers, demonstrating that the cells have undergone EMT, indicates that mesenchymal properties, typical for aggressive single cell migration, are also necessary for the observed collective invasion.

In contrast to cancer cell aggregates, isolated cancer cells exhibited both up- and downstream migration when subjected to IFF in a compartmentalized chip, and these migration patterns depended on the cell density (Polacheck et al., 2011). This dependence could be explained by a competition between tensional cues from ECM adhesions that induce upstream migration and autologous chemotaxis, which induces downstream migration. In the latter case, an isolated cell is attracted to its own growth factors being carried downstream the IFF (Polacheck et al., 2014). This local chemotactic gradient disappeared when cell numbers were increased, leading to more upstream cell migration driven by the competing tensional cues. In other work, the different cellular subpopulations that migrate either upstream or downstream the IFF could be identified by applying single cell tracking inside compartmentalized chips (Haessler et al., 2012). Moreover, a relationship between IFF and the migration mode of cancer cells was found: when subjected to IFF, an increased number of cells exhibited amoeboid migration, with fewer exhibiting mesenchymal migration (Huang et al., 2015; Box 1), indicating that isolated cancer cells might be driven towards a less mesenchymal phenotype, as opposed to cell aggregates. These results imply that isolated cancer cells migrate, and thus metastasize, differently than cancer cell aggregates do. Although the relevance of single versus collective invasion is not completely clear, insights into the mechanisms that underlie these types of invasion directed by IFP gradients could lead to more targeted therapeutic approaches to prevent metastasis.

### Mechanical tissue deformation

To our knowledge, only two CoC-based reports on the integration of physiological mechanical tissue deformation have been published. Huang et al. studied the interaction between fibroblasts and lung cancer cells in a compartmentalized microfluidic chip, in which cancer cells were supplied with conditioned growth medium from the fibroblast-containing chamber (Huang et al., 2013). By periodically stretching the fibroblast culture surface, which mimicked the tensile strain that lungs are subjected to during breathing cycles, the migration speed of the lung cancer cells was significantly reduced, showing that tensile stress influences fibroblasts, whose secretome in turn affects the behavior of lung cancer cells. In a recent study, non-small-cell lung cancer cells were included in a lung-on-a-chip organ model that included both the epithelial cell layer, EC layer and physiological periodic strain (Hassell et al., 2017). Reduced invasion was observed in the dynamically stretched versus the static samples, and the development of resistance to tyrosine kinase inhibitor was observed in the dynamic, but not in the static, model. These results indicate that mechanical deformation can affect both cancer cell invasion and therapeutic resistance.

## The future of cancer-on-a-chip technology

As discussed in the previous sections, CoC approaches have been used to answer many questions about the influence of the TME on cancer metastasis, but they have also opened up new questions and pointed towards new avenues of research. Here, we take a closer look at these questions and possible research directions. To tackle these questions, researchers must choose the most appropriate chip design. For this purpose, we provide an overview of the current CoC literature in Table S1.

Most biochemical cue-oriented CoC studies have focused on oxygen, and some analyzed acidity and lactate levels. Such research has indicated that aerotaxis is a relevant mechanism in cancer cell migration, and that acid and lactate gradients play a role in directing cancer cell invasion. Our understanding of these effects is far from complete, but the CoC methods discussed here are promising tools to investigate the effects of these and possibly other biochemical gradients on cancer cells. Importantly, the contributions of these biochemical cues should be evaluated in combination with different matrices and TME cell types, as we have seen that many of these effects are influenced by TME factors, and not only by the cancer cells. A striking example is how hypoxia can both directly induce invasion but can also indirectly activate CAFs and MMPs to drive the ECM remodeling that facilitates invasion.

Many kinds of cell–cell interactions have been studied in CoC devices. In the near future, they could be applied to obtain additional insight into the mechanisms underlying the recruitment and activation of both CAFs and TAMs. Moreover, the role of the M1/M2 phenotype of TAMs, the relevance of CAF subpopulations or the extent to which CAFs influence other stromal cells could be studied. Other possible experiments could be tailored towards investigating the relatively new concept of angiocrine signaling and to study the interaction mechanisms between ECs and cancer cells. Similarly, the relevance of intravasation into lymphatic vessels should be investigated in more detail.

At this point, it is important to note that the list of different TME cell types discussed in this article is by no means exhaustive; many more cell types, such as mesenchymal stem cells (Ma et al., 2012), natural killer cells (Ayuso et al., 2016), dendritic cells (Parlato et al., 2017) and adipocytes play a role in invasion and intravasation, and their roles could also be (or are being) studied in a CoC setting. The main challenge, however, is that the relative contribution of an individual cell type is difficult to evaluate, as many can interact with each other and synergistically activate the cancer cells. Future CoC work should therefore focus on understanding and evaluating these types of cell–cell interactions.

The ECM has been studied to some extent in CoC systems, mainly focusing on the effect of ECM changes during invasion. In future work, CoC models could be used to further increase our understanding of how the mechanical properties and architecture of both the ECM and the BM affect invasion. This could be enabled by patterning ECM and BM components with different (mechanical) properties on a chip that facilitates control over cues, such as chemotactic gradients and the cell types involved in matrix remodeling. By also varying the matrix composition, more insight could be generated into the role of different ECM constituents in directing cellular behavior.

The CoC work on mechanical cues has mostly focused on interstitial pressure and flow as drivers of cancer cell migration. The literature reviewed here demonstrates how the integration of more conventional read-outs could lead to novel mechanistic insights, such as the competition between autologous chemotaxis and matrix-mediated cancer cell migration. However, the integration of organ-level mechanical cues in CoC systems is clearly still in its infancy. Most CoC devices are still relatively static, while many organs, such as the lung, colon and stomach, are highly dynamic. Here, the CoC field can learn from the broader field of organ-on-a-chip, in which this type of mechanical cue has been integrated in many different organ models (reviewed in Ingber, 2016).

We have seen that CoC models are an enabling technology for quantitative analysis of the roles of the different TME cues in metastasis. However, evaluating the synergy between these cues in CoC chips, with the added complexity of *in vivo* like cross-talk, is still a major challenge. Here, the field of CoC could benefit from more advanced theoretical modeling, which could lead to extremely powerful approaches to study the roles of the TME in cancer metastasis.

## Conclusion

We have highlighted how different cues from the TME can affect the onset of metastasis, and we have reviewed the most recent CoC developments showing how these models can help decipher the complex interplay within and between the cancer cells and the TME. Furthermore, we have highlighted remaining challenges for which these promising technologies could be used to overcome. In a much broader perspective, the technologies developed for CoC models are not limited to studying cancer invasion and the TME alone. Here, we focused on the onset of metastasis, but CoC technology can be, and is, applied to study other steps in the process, such as extravasation (Jeon et al., 2015). Whether used to study the full metastatic cascade or its onset alone, CoC technology has the potential to reduce our reliance on animal models as a complementary research tool. Beyond generating mechanistic insight in the metastatic cascade, CoC models could be combined with clinical material to investigate patient-specific cancer progression. This could drastically change the way we can test drug efficacy, or even develop new therapies to specifically prevent metastasis.

## Supplementary Material

Supplementary information
