# Supplementary material for: Metastasis in context: modeling the tumor microenvironment with cancer-on-a-chip approaches
Source: Dis Model Mech. 2018 Mar 1;11(3):dmm033100. doi: 10.1242/dmm.033100 (PMC5897732; doi:10.1242/dmm.033100)
Supplement: Supplementary information [file dmm-11-033100-s1.pdf]

Biochemical cues

Chip type	TME cue	Reference	Flow control	Live imaging	small volume	Co-culture
2D chip	Oxygen, drugs	(Wang et al., 2013)	+	++	+	
	Oxygen, drugs, chemoattractant	(Chang et al., 2014)	++	++		
	Oxygen, drugs	(Wang et al., 2015a)		+	+	
	Oxygen	(Zhang et al., 2015)	++	++	++	
	Oxygen	(Yahara et al., 2016)		+	+	
	Oxygen	(Morshed and Dutta, 2017)	+		+	
Compart-mentalized chip	Nutrients	(Walsh et al., 2009)	++	++	+	
	Oxygen	(Funamoto et al., 2012)	+	++	++	
	Oxygen	(Acosta et al., 2014)	+	+	++	
Membrane chip	Oxygen	(Oppegard and Eddington, 2013)	+		+	
	Oxygen, drugs	(Peng et al., 2013)	++		+	
	Nutrients	(Yan et al., 2013)	+		+	

Advantages of CoC approach: Flow control, Live imaging, small volume, Co-culture

Biochemical cues & Cellular environment

Chip type	TME cue	Reference	Flow control	Live imaging	small volume	Co-culture
2D chip	Acidity, fibroblasts	(Xu et al., 2015)				++
Compart-mentalized chip	Oxygen, drugs, natural killer cells	(Ayuso et al., 2016)	++	++		++

Advantages of CoC approach: Flow control, Live imaging, small volume, Co-culture

Cellular environment

Chip type	TME cue	Reference	Flow control	Live imaging	small volume	Co-culture
2D chip	Fibroblasts	(Ma et al., 2010)		++		++
	Fibroblasts	(Hsu et al., 2011)	++	+		++
	Macrophages, fibroblasts	(Hsu et al., 2012)	++			++
	Leukocytes	(Agliari et al., 2014)		++		++
	Fibroblasts	(Yu et al., 2016)	+	++		++
	Endothelial cells	(Zhu et al., 2016)		++		++
	Dendritic cells	(Parlato et al., 2017)	++	++		++
Lumen chip	Endothelial cells	(Wang et al., 2015b)		+		++
Compart-mentalized chip (part 1)	Fibroblasts	(Liu et al., 2010)			+	++
	Mesenchymal stem cells	(Ma et al., 2012)		++		++
	Endothelial cells	(Zervantonakis et al., 2012)	++	++		++
	Endothelial cells, osteo-cells	(Bersini et al., 2014)		+		++

Advantages of CoC approach: Flow control, Live imaging, small volume, Co-culture

Chip type	TME cue	Reference	Flow control	Live imaging	small volume	Co-culture
Compart-mentalized chip (part 2)	Endothelial cells, fibroblasts	(Lee et al., 2014)	+			++
	Macrophages	(Zhao et al., 2015)		+		++
	Macrophages	(Bai et al., 2015)		++	+	++
	Fibroblasts	(Li et al., 2016)		++		++
Lumen-compart-mentalized chip	Fibroblasts, mammary epithelial cells	(Bischel et al., 2015)	++		+	++
Membrane chip	Fibroblasts, mammary epithelial cells	(Choi et al., 2015)	++	+		++
	Lymphatic endothelial cells	(Pisano et al., 2015)	++			++

Cellular environment & ECM

Advantages of CoC approach

Chip type	TME cue	Reference	Flow control	Live imaging	small volume	Co-culture
Lumen chip	Endothelial cells, microtracks	(Wong and Searson, 2014)	++	++		++
Compart-mentalized chip	Macrophages, hydrogel pattern	(Huang et al., 2009)		++		++
	Fibroblasts, hydrogel pattern	(Truong et al., 2016)	+	++		++
	Fibroblasts, hydrogel pattern	(Gioiella et al., 2016)	++	++	+	++
Y chip	Fibroblasts, hydrogel pattern	(Sung et al., 2011)	++	++	+	++
	Pancreatic stellate cells, hydrogel pattern	(Drifka et al., 2013)	++	++	+	++

ECM

Advantages of CoC approach

Chip type	TME cue	Reference	Flow control	Live imaging	small volume	Co-culture
Compart-mentalized chip	Matrix architecture	(Chaw et al., 2007a)		++	+	
	Matrix architecture	(Chaw et al., 2007b)		++	+	
	Matrix composition	(Shin et al., 2014)		++		+
	Matrix architecture	(Han et al., 2016)	++	++		
	Matrix architecture, mechanics	(Anguiano et al., 2017)		++		
Membrane chip	Matrix architecture	(Eslami Amirabadi et al., 2017)			++	+

Disease Models & Mechanisms • Supplementary information

Mechanical cues

Advantages of CoC approach

Chip type	TME cue	Reference	Flow control	Live imaging	small volume	Co-culture
2D chip	Fibroblasts, Cyclic tensile strain	(Huang et al., 2013)	++	++	+	++
Lumen chip	Interstitial fluid pressure	(Tien et al., 2012)	++	+		
	Interstitial fluid pressure	(Piotrowski-Daspit et al., 2016)	++	++		
Compart-mentalized chip	Interstitial fluid flow	(Polacheck et al., 2011)	++	++	+	
	Interstitial fluid flow	(Haessler et al., 2012)	++	++		
	Interstitial fluid flow, drugs	(Kalchman et al., 2013)	++	+	+	++
	Interstitial fluid flow	(Polacheck et al., 2014)	++	++	+	
	Interstitial fluid flow	(Huang et al., 2015)	++	++	+	
Membrane chip	Epithelial cells, Endothelial cells, Cyclic tensile strain	(Hassell et al., 2017)	+	++	+	++

References

Acosta, M. A., Jiang, X., Huang, P.-K., Cutler, K. B., Grant, C. S., Walker, G. M. and Gamcsik, M. P. (2014). A microfluidic device to study cancer metastasis under chronic and intermittent hypoxia. *Biomicrofluidics* **8**, 54117.

Agliari, E., Biselli, E., De Ninno, A., Schiavoni, G., Gabriele, L., Gerardino, A., Mattei, F., Barra, A. and Businaro, L. (2014). Cancer-driven dynamics of immune cells in a microfluidic environment. *Sci. Rep.* **4**, 6639.

Anguiano, M., Castilla, C., Maška, M., Ederra, C., Peláez, R., Morales, X., Muñoz-Arrieta, G., Mujika, M., Kozubek, M., Muñoz-Barrutia, A., et al. (2017). Characterization of three-dimensional cancer cell migration in mixed collagen-Matrigel scaffolds using microfluidics and image analysis. *PLoS One* **12**, e0171417.

Ayuso, J. M., Virumbrales-Muñoz, M., Lacueva, A., Lanuza, P. M., Checa-Chavarria, E., Botella, P., Fernández, E., Doblare, M., Allison, S. J., Phillips, R. M., et al. (2016). Development and characterization of a microfluidic model of the tumour microenvironment. *Sci. Rep.* **6**, 36086.

Bai, J., Adriani, G., Dang, T., Tu, T., Penny, H. L., Wong, S., Kamm, R. D. and Thiery, J.-P. (2015). Contact-dependent carcinoma aggregate dispersion by M2a macrophages via ICAM-1 and β2 integrin interactions. *Oncotarget* **6**, 25295–25307.

Bersini, S., Jeon, J. S., Dubini, G., Arrigoni, C., Chung, S., Charest, J. L., Moretti, M. and Kamm, R. D. (2014). A microfluidic 3D in vitro model for specificity of breast cancer metastasis to bone. *Biomaterials* **35**, 2454–2461.

Bischel, L. L., Beebe, D. J. and Sung, K. E. (2015). Microfluidic model of ductal carcinoma in situ with 3D, organotypic structure. *BMC Cancer* **15**, 12.

Chang, C.-W., Cheng, Y.-J., Tu, M., Chen, Y.-H., Peng, C.-C., Liao, W.-H. and Tung, Y.-C. (2014). A polydimethylsiloxane–polycarbonate hybrid microfluidic device capable of generating perpendicular chemical and oxygen gradients for cell culture studies. *Lab Chip* **14**, 3762–3772.

Chaw, K. C., Manimaran, M., Tay, E. H. and Swaminathan, S. (2007a). Multi-step microfluidic device for studying cancer metastasis. *Lab Chip* **7**, 1041.

Chaw, K. C., Manimaran, M., Tay, F. E. H. and Swaminathan, S. (2007b). Matrigel coated polydimethylsiloxane based microfluidic devices for studying metastatic and non-metastatic cancer cell invasion and migration. *Biomed. Microdevices* **9**, 597–602.

Choi, Y., Hyun, E., Seo, J., Blundell, C., Kim, H. C., Lee, E., Lee, S. H., Moon, A., Moon, W. K. and Huh, D. (2015). A microengineered pathophysiological model of early-stage breast cancer. *Lab a Chip - Miniaturisation Chem. Biol.* **15**, 3350–3357.

Drifka, C. R., Eliceiri, K. W., Weber, S. M. and Kao, W. J. (2013). A bioengineered heterotypic stroma–cancer microenvironment model to study pancreatic ductal adenocarcinoma. *Lab Chip* **13**, 3965–3975.

Eslami Amirabadi, H., SahebAli, S., Frimat, J. P., Luttge, R. and den Toonder, J. M. J. (2017). A novel method to understand tumor cell invasion: integrating extracellular matrix mimicking layers in microfluidic chips by "selective curing." *Biomed. Microdevices* **19**, 92.

Funamoto, K., Zervantonakis, I. K., Liu, Y., Ochs, C. J., Kim, C. and Kamm, R. D. (2012). A novel microfluidic platform for high-resolution imaging of a three-dimensional cell culture under a controlled hypoxic environment. *Lab Chip* **12**, 4855–4863.

Gioiella, F., Urciuolo, F., Imparato, G., Brancato, V. and Netti, P. A. (2016). An Engineered Breast Cancer Model on a Chip to Replicate ECM-Activation In Vitro during Tumor Progression. *Adv. Healthc. Mater.* **5**, 3074–3084.

Haessler, U., Teo, J. C. M., Foretay, D., Renaud, P. and Swartz, M. a. (2012). Migration dynamics of breast cancer cells in a tunable 3D interstitial flow chamber. *Integr. Biol.* **4**, 401–409.

Han, W., Chen, S., Yuan, W., Fan, Q., Tian, J., Wang, X., Chen, L., Zhang, X., Wei, W., Liu, R., et al. (2016). Oriented collagen fibers direct tumor cell intravasation. *Proc. Natl. Acad. Sci.* **113**, 11208–11213.

Hassell, B. A., Goyal, G., Lee, E., Sontheimer-Phelps, A., Levy, O., Chen, C. S. and Ingber, D. E. (2017). Human Organ Chip Models Recapitulate Orthotopic Lung Cancer Growth, Therapeutic Responses, and Tumor Dormancy In Vitro. *Cell Rep.* **21**, 508–516.

Hsu, T.-H., Xiao, J.-L., Tsao, Y.-W., Kao, Y.-L., Huang, S.-H., Liao, W.-Y. and Lee, C.-H. (2011). Analysis of the paracrine loop between cancer cells and fibroblasts using a microfluidic chip. *Lab Chip* **11**, 1808–1814.

Hsu, T.-H., Kao, Y.-L., Lin, W.-L., Xiao, J.-L., Kuo, P.-L., Wu, C.-W., Liao, W.-Y. and Lee, C.-H. (2012). The migration speed of cancer cells influenced by macrophages and myofibroblasts co-cultured in a microfluidic chip. *Integr. Biol.* **4**, 177–182.

Huang, C. P., Lu, J., Seon, H., Lee, A. P., Flanagan, L. a, Kim, H.-Y., Putnam, A. J. and Jeon, N. L. (2009). Engineering microscale cellular niches for three-dimensional multicellular co-cultures. *Lab Chip* **9**, 1740.

Huang, J.-W., Pan, H.-J., Yao, W.-Y., Tsao, Y.-W., Liao, W.-Y., Wu, C.-W., Tung, Y.-C. and Lee, C.-H. (2013). Interaction between lung cancer cell and myofibroblast influenced by cyclic tensile strain. *Lab Chip* **13**, 1114–1120.

Huang, Y. L., Tung, C.-K., Zheng, A., Kim, B. J. and Wu, M. (2015). Interstitial flows promote amoeboid over mesenchymal motility of breast cancer cells revealed by a three dimensional microfluidic model. *Integr. Biol.* **7**, 1402–1411.

Kalchman, J., Fujioka, S., Chung, S., Kikkawa, Y., Mitaka, T., Kamm, R. D., Tanishita, K. and Sudo, R. (2013). A three-dimensional microfluidic tumor cell migration assay to screen the effect of anti-migratory drugs and interstitial flow. *Microfluid. Nanofluidics* **14**, 969–981.

Lee, H., Park, W., Ryu, H. and Jeon, N. L. (2014). A microfluidic platform for quantitative analysis of cancer angiogenesis and intravasation. *Biomicrofluidics* **8**, 54102.

Li, J., Jia, Z., Kong, J., Zhang, F., Fang, S., Li, X., Li, W., Yang, X., Luo, Y., Lin, B., et al. (2016). Carcinoma-associated fibroblasts lead the invasion of salivary gland adenoid cystic carcinoma cells by creating an invasive track. *PLoS One* **11**, 1–15.

Liu, T., Lin, B. and Qin, J. (2010). Carcinoma-associated fibroblasts promoted tumor spheroid invasion on a microfluidic 3D co-culture device. *Lab Chip* **10**, 1671.

Ma, H., Liu, T., Qin, J. and Lin, B. (2010). Characterization of the interaction between fibroblasts and tumor cells on a microfluidic co-culture device. *Electrophoresis* **31**, 1599–1605.

Ma, H., Zhang, M. and Qin, J. (2012). Probing the role of mesenchymal stem cells in salivary gland cancer on biomimetic microdevices. *Integr. Biol.* **4**, 522–530.

Morshed, A. and Dutta, P. (2017). Hypoxic behavior in cells under controlled microfluidic environment. *Biochim. Biophys. Acta - Gen. Subj.* **1861**, 759–771.

Oppegard, S. C. and Eddington, D. T. (2013). A microfabricated platform for establishing oxygen gradients in 3-D constructs. *Biomed. Microdevices* **15**, 407–414.

Parlato, S., De Ninno, A., Molfetta, R., Toschi, E., Salerno, D., Mencattini, A., Romagnoli, G., Fragale, A., Roccazzello, L., Buoncervello, M., et al. (2017). 3D Microfluidic model for evaluating immunotherapy

efficacy by tracking dendritic cell behaviour toward tumor cells. *Sci. Rep.* **7**, 1093.

Peng, C.-C., Liao, W.-H., Chen, Y.-H., Wu, C.-Y. and Tung, Y.-C. (2013). A microfluidic cell culture array with various oxygen tensions. *Lab Chip* **13**, 3239.

Piotrowski-Daspit, A. S., Tien, J. and Nelson, C. M. (2016). Interstitial fluid pressure regulates collective invasion in engineered human breast tumors via Snail, vimentin, and E-cadherin. *Integr. Biol.* **8**, 319–331.

Pisano, M., Triacca, V., Barbee, K. A. and Swartz, M. A. (2015). An in vitro model of the tumor-lymphatic microenvironment with simultaneous transendothelial and luminal flows reveals mechanisms of flow enhanced invasion. *Integr. Biol.* **7**, 525–533.

Polacheck, W. J., Charest, J. L. and Kamm, R. D. (2011). Interstitial flow influences direction of tumor cell migration through competing mechanisms. *Proc. Natl. Acad. Sci.* **108**, 11115–11120.

Polacheck, W. J., German, A. E., Mammoto, A., Ingber, D. E. and Kamm, R. D. (2014). Mechanotransduction of fluid stresses governs 3D cell migration. *Proc. Natl. Acad. Sci.* **111**, 2447–2452.

Shin, Y., Han, S., Chung, E. and Chung, S. (2014). Intratumoral phenotypic heterogeneity as an encourager of cancer invasion. *Integr. Biol.* **6**, 654–661.

Sung, K. E., Yang, N., Pehlke, C., Keely, P. J., Eliceiri, K. W., Friedl, A. and Beebe, D. J. (2011). Transition to invasion in breast cancer: a microfluidic in vitro model enables examination of spatial and temporal effects. *Integr. Biol.* **3**, 439–450.

Tien, J., Truslow, J. G. and Nelson, C. M. (2012). Modulation of Invasive Phenotype by Interstitial Pressure-Driven Convection in Aggregates of Human Breast Cancer Cells. *PLoS One* **7**, e45191.

Truong, D., Puleo, J., Llave, A., Mouneimne, G., Kamm, R. D. and Nikkhah, M. (2016). Breast Cancer Cell Invasion into a Three Dimensional Tumor-Stroma Microenvironment. *Sci. Rep.* **6**, 34094.

Walsh, C. L., Babin, B. M., Kasinskas, R. W., Foster, J. A., McGarry, M. J. and Forbes, N. S. (2009). A multipurpose microfluidic device designed to mimic microenvironment gradients and develop targeted cancer therapeutics. *Lab Chip* **9**, 545–554.

Wang, L., Liu, W., Wang, Y., Wang, J., Tu, Q., Liu, R. and Wang, J. (2013). Construction of oxygen and chemical concentration gradients in a single microfluidic device for studying tumor cell-drug interactions in a dynamic hypoxia microenvironment. *Lab Chip* **13**, 695–705.

Wang, Z., Liu, Z., Li, L. and Liang, Q. (2015a). Investigation into the hypoxia-dependent cytotoxicity of anticancer drugs under oxygen gradient in a microfluidic device. *Microfluid. Nanofluidics* **19**, 1271–1279.

Wang, X.-Y., Pei, Y., Xie, M., Jin, Z., Xiao, Y., Wang, Y., Zhang, L., Li, Y. and Huang, W. (2015b). An artificial blood vessel implanted three-dimensional microsystem for modeling transvascular migration of tumor cells. *Lab Chip* **15**, 1178–1187.

Wong, A. D. and Searson, P. C. (2014). Live-Cell Imaging of Invasion and Intravasation in an Artificial Microvessel Platform. *Cancer Res.* **74**, 4937–4945.

Xu, X.-D., Shao, S.-X., Cao, Y.-W., Yang, X.-C., Shi, H.-Q., Wang, Y.-L., Xue, S.-Y., Wang, X.-S. and Niu, H.-T. (2015). The study of energy metabolism in bladder cancer cells in co-culture conditions using a microfluidic chip. *Int. J. Clin. Exp. Med.* **8**, 12327–12336.

Yahara, D., Yoshida, T., Enokida, Y. and Takahashi, E. (2016). Directional Migration of MDA-MB-231 Cells Under Oxygen Concentration Gradients. In *Advances in Experimental Medicine and Biology*, pp. 129–134.

Yan, W., Zhang, Q., Chen, B., Liang, G. T., Li, W. X., Zhou, X. M. and Liu, D. Y. (2013). Study on microenvironment acidification by microfluidic chip with multilayer-paper supported breast cancer tissue. *Fenxi Huaxue/ Chinese J. Anal. Chem.* **41**, 822–827.

Yu, T., Guo, Z., Fan, H., Song, J., Liu, Y., Gao, Z. and Wang, Q. (2016). Cancer-associated fibroblasts promote non-small cell lung cancer cell invasion by upregulation of glucose-regulated protein 78 (GRP78) expression in an integrated bionic microfluidic device. *Oncotarget* **7**, 25593–25603.

Zervantonakis, I. K., Hughes-Alford, S. K., Charest, J. L., Condeelis, J. S., Gertler, F. B. and Kamm, R. D. (2012). Three-dimensional microfluidic model for tumor cell intravasation and endothelial barrier function. *Proc. Natl. Acad. Sci.* **109**, 13515–13520.

Zhang, Y., Wen, J., Zhou, L. and Qin, L. (2015). Utilizing a high-throughput microfluidic platform to study hypoxia-driven mesenchymal-mode cell migration. *Integr. Biol.* **7**, 672–680.

Zhao, Y., Wang, D., Xu, T., Liu, P., Cao, Y., Wang, Y., Yang, X., Xu, X., Wang, X. and Niu, H. (2015). Bladder cancer cells re-educate TAMs through lactate shuttling in the microfluidic cancer microenvironment. *Oncotarget* **6**, 39196–39210.

Zhu, G., Wang, D., Li, S., Yang, X., Cao, Y., Wang, Y. and Niu, H. (2016). Acute effect of lactic acid on tumor-endothelial cell metabolic coupling in the tumor microenvironment. *Oncol. Lett.* **12**, 3478–3484.
